# Serum Metabolomics of Burkitt Lymphoma Mouse Models

**DOI:** 10.1371/journal.pone.0170896

**Published:** 2017-01-27

**Authors:** Fengmin Yang, Jie Du, Hong Zhang, Guorui Ruan, Junfeng Xiang, Lixia Wang, Hongxia Sun, Aijiao Guan, Gang Shen, Yan Liu, Xiaomeng Guo, Qian Li, Yalin Tang

**Affiliations:** 1 National Laboratory for Molecular Sciences, Center for Molecular Sciences, State Key Laboratory for Structural Chemistry of Unstable and Stable Species, Institute of Chemistry, Chinese Academy of Sciences, Beijing, P. R. China; 2 JOINN Laboratories, Inc., Beijing, P. R. China; 3 Peking University People’s Hospital and Institute of Hematology, Beijing, P. R. China; 4 University of the Chinese Academy of Sciences, Beijing, P. R. China; University of South Alabama Mitchell Cancer Institute, UNITED STATES

## Abstract

Burkitt lymphoma (BL) is a rare and highly aggressive type of non-Hodgkin lymphoma. The mortality rate of BL patients is very high due to the rapid growth rate and frequent systemic spread of the disease. A better understanding of the pathogenesis, more sensitive diagnostic tools and effective treatment methods for BL are essential. Metabolomics, an important aspect of systems biology, allows the comprehensive analysis of global, dynamic and endogenous biological metabolites based on their nuclear magnetic resonance (NMR) and mass spectrometry (MS). It has already been used to investigate the pathogenesis and discover new biomarkers for disease diagnosis and prognosis. In this study, we analyzed differences of serum metabolites in BL mice and normal mice by NMR-based metabolomics. We found that metabolites associated with energy metabolism, amino acid metabolism, fatty acid metabolism and choline phospholipid metabolism were altered in BL mice. The diagnostic potential of the metabolite differences was investigated in this study. Glutamate, glycerol and choline had a high diagnostic accuracy; in contrast, isoleucine, leucine, pyruvate, lysine, α-ketoglutarate, betaine, glycine, creatine, serine, lactate, tyrosine, phenylalanine, histidine and formate enabled the accurate differentiation of BL mice from normal mice. The discovery of abnormal metabolism and relevant differential metabolites may provide useful clues for developing novel, noninvasive approaches for the diagnosis and prognosis of BL based on these potential biomarkers.

## Introduction

BL is a rare and highly aggressive type of non-Hodgkin lymphoma, mainly from B lymphocytes, that was first discovered by British surgeon Dennis Burkitt [1]. Currently, BL is divided into three subtypes: endemic, sporadic and HIV-associated. It is induced by Epstein-Barr (EB) virus infection and *c-myc* gene overexpression [2–4]. In malaria-prevalent regions of equatorial Africa, children aged 4–7 years are very susceptible to BL, often involving the mandible and kidney. It can also affect the ileum, cecum, ovaries and breast [5]. In Western countries, approximately 1% to 2% of adult lymphoma patients have BL [6], and approximately 30% to 50% of childhood lymphoma patients also have BL [7]. Although rare, BL exhibits a rapid growth rate and frequent systemic spread, which accounts for 70% to 80% of patients presenting at advanced stages of disease at the time of diagnosis. Surgery and chemotherapy are less effective in adult BL. The mortality rate of BL is very high for these reasons. Hence, a better understanding of the pathogenesis, more sensitive diagnostic tools and effective treatment methods for BL are essential.

Metabolomics is an important aspect of systems biology that can comprehensively analyze global, dynamic and endogenous biological metabolites based on NMR or MS [8]. Metabolomics has already been used to investigate the pathogenesis and discover new biomarkers for disease diagnosis and prognosis. Brindle et al. demonstrated that metabolomics can accurately, noninvasively and rapidly diagnose coronary heart disease by NMR [9]. Using metabolomics, Sreekurnar et al. found that sarcosine is an important biomarker in prostate cancer [10]. Denkert found that many metabolites were different between normal colon and colorectal cancer tissues [11]. Huang et al. discovered that the combination of betaine and propionylcarnitine may be used as a diagnostic biomarker for hepatocellular carcinoma, using nontargeted tissue metabolomics [12]. Therefore, metabolomics can be used not only to discover new biomarkers but also to develop noninvasive, potentially diagnostic and prognostic tools.

Metabolomics research using clinical serum samples faces many challenges because the concentrations of metabolites vary frequently due to various genetic and environmental factors. In addition, serum samples from newly diagnosed BL patients may not be readily available. Li Zhang [13] and Tobias Weber [14] both established BL mouse disease models by implanting human Raji cells into mice to study the therapeutic effect and mechanism of targeted delivery against BL. Wen Lian Chen [15] investigated the activity of fructose utilization and the therapeutic potential of inhibitors of related metabolic pathways using an AML mouse model. François Jouret [16] established a mouse model of ischemia/reperfusion and carried out metabolomics using urine, serum and kidney samples. Leila Pirhaji [17] established a Huntington disease mouse model and demonstrated a new network-based approach by studying the metabolomics of the model. Therefore, many similarities exist between mouse metabolism and human metabolism. The serum metabolomics of BL mouse models implanted with human Raji cells could provide important insight into the clinical diagnosis and treatment of BL.

Currently, little is known about the metabolomics of BL. The comprehensive pathogenesis of BL is expected to be revealed by metabolomics, which is very important for the diagnosis and treatment of BL. In this study, we analyzed serum metabolomics of BL mouse models, based on NMR techniques. The concentration of some serum metabolites such as glucose, glutamate, and unsaturated lipids was significantly different between BL mice and wild-type mice. Abnormality of metabolism and the relevant different metabolites of BL were discovered. These results may provide useful clues for developing novel noninvasive methods for the diagnosis and prognosis of BL based on these potential biomarkers.

## Materials and Methods

### Animals and sample collections

Twenty non-obese diabetic-severe combined immune-deficiency (NOD-SCID) mice (20 to 26 g) aged seven to nine weeks were housed in cages under a regular light cycle (12 h) and fed a sterilized mouse diet and water. Ten mice served as controls, and the others were tumor-bearing. Raji cells (2 × 10^6^ cells / mouse) were injected subcutaneously into the right front axilla of the mice. The samples were collected when the tumor volume had reached approximately 500 to 1000 mm^3^. This study was performed in strict accordance with the recommendations of the Guidelines for the Care and Use of Laboratory Animals of the National Science Center of China. The protocol was approved by the Committee on the Ethics of Animal Experiments of China. The animals were housed and cared for in accordance with the guidelines established by the National Science Center of China.

All blood samples without anticoagulants were placed at room temperature for 45 minutes and centrifuged at 8,000 g for 10 min at 4°C. The serum samples were collected and stored at -80°C until analysis.

### Sample preparation

The serum samples were thawed at room temperature and centrifuged at 12,000 g for 10 min at 4°C. Each serum sample (100 μL) was mixed with phosphate buffer (100 μL) (1:1 v/v, mixture of 1 M K_2_HPO_4_ and 0.25 M NaH_2_PO_4_; pH 7.4, 100% D_2_O) by vortexing for 1 min and centrifuged at 12,000 g for 10 min at 4°C. Next, 150 μL of the supernatant was transferred to the 3 mm NMR tube.

### ^1^H NMR spectroscopy of serum samples

The serum samples were analyzed by ^1^H NMR spectroscopy at 600.13 MHz in a Bruker AVANCE 600 spectrometer equipped with a TBO probe. To detect the low molecular weight components of serum, the ^1^H NMR spectra of all serum samples were acquired at 300 K using standard Carr-Purcell-Meiboom-Gill (CPMG) plus sequence ((RD-90°-(ô-180°-ô) _n_ -acquire)) with a total spin—spin relaxation delay of 40 ms [18]. The water signal was eliminated with the presaturation sequence, and the relaxation delay was 2 s. Data points were adjusted to 64 K with a spectral width of 30 ppm. The number of scans was set to 256. The free induction delays were multiplied by an exponential line-broadening factor of 1.0 Hz before Fourier transformation. ^1^H chemical shifts were referenced internally to the proton signal of lactate at chemical shift (δ) 1.34. Furthermore, diffusion-edited experiments were also carried out with bipolar pulse pair-longitudinal eddy current delay pulse sequence (BPP-LED) (RD-90°-G1-180°-G1-90°-G2-D-90°-G1-180°-G1-90°-G2-t-90°-acquire) to detect the lipids of serum [19]. The gradient amplitude was set at 50.0 G/cm, with a diffusion delay of 50 ms. Data points were collected with a spectral width of 30 ppm. A line-broadening factor of 1.0 Hz was applied to the free induction delays prior to Fourier transformation. ^1^H chemical shifts were referenced internally to the proton signal of phosphatidylcholine at chemical shift (δ) 3.22.

To identify the metabolites, two-dimensional NMR spectra were acquired, including ^1^H-^1^H correlation spectroscopy (COSY), total correlation spectroscopy (TCOSY), ^1^H-^13^C heteronuclear single quantum correlation (HSQC) and ^1^H-^13^C heteronuclear multiple bond correlation (HMBC) spectra.

### NMR data processing and multivariate analysis

All serum ^1^H NMR spectra were corrected for phase and baseline using MestReNova (6.1.1-6384-Win). The spectra over the range of δ 0.5–9.5 for CPMG were divided into buckets with equal width of 0.005 ppm, and the regions at δ 4.200–5.200 were excluded for eliminating the interference of the water signals. The spectra over the range of δ 0.5–9.5 for BPP-LED were divided into buckets with equal width of 0.005 ppm, and the regions at δ 4.385–5.075 were excluded for eliminating the interference of the water signals. Buckets were normalized to a constant sum (100) of all spectra intensity to reduce the differences of the concentration between the serum samples [20].

The data were imported into SIMCA 14 Umetrics for multivariate analysis. The principal component analysis (PCA) was used to discern the presence of inherent similarities of spectral profiles. The partial least squares-discriminant analysis (PLS-DA) or the orthogonal partial least squares-discriminant analysis (OPLS-DA) was used to find the metabolite differences between the control and tumor-bearing mice [21]. The PCA and PLS-DA of CPMG data were only pareto-scaled, while the PCA and OPLS-DA of BPP-LED data were mean-centered and pareto-scaled scaled before analysis, respectively. The quality of each model was determined by the goodness of fit parameter (R2) and a goodness of prediction parameter (Q2) [22]. Two components were reserved to calculate the score contribution weights to determine the variables which were responsible for the separation between groups. To validate the model, CV-ANOVA method [23,24] and permutation testing (n = 200) were implemented. p-values less than 0.05 were considered to indicate statistically significant differences using the non-parametric Mann-Whitney test. To test the clustering and diagnostic potential of metabolite differences, the hierarchical cluster analysis (HCA, Ri386 3.2.1) and the receiver operating characteristic curve (ROC, SPSS 17.0) were conducted.

## Results

### ^1^H NMR spectra of serum samples

**Fig 1:**
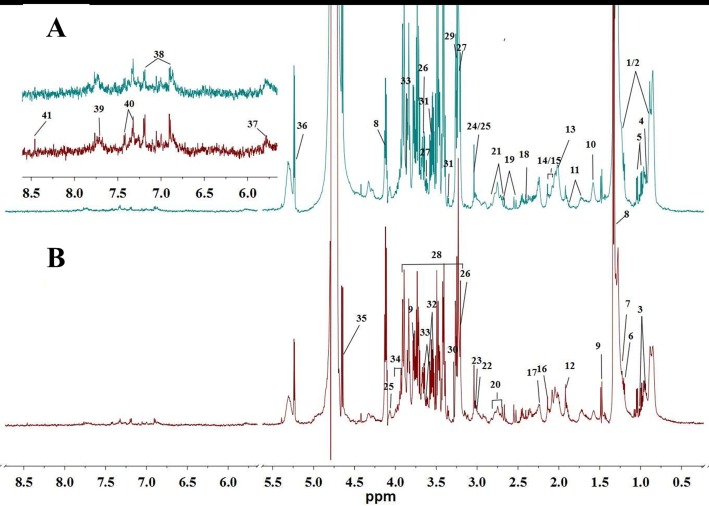
Typical 600 MHz ^1^H CPMG spectra of serum samples. (A) controls (B) tumor-bearing mice. Keys for metabolites: 1, Lipids (mainly LDL); 2, Lipids (mainly VLDL); 3, Isoleucine; 4, Leucine; 5, Valine; 6, 3-Hydroxybutyrate; 7, Unknown; 8, Lactate; 9, Alanine; 10, Citrulline; 11, Arginine; 12, Acetate; 13, Proline; 14, Glutamate; 15, Glutamine; 16, Methionine; 17, Lipid; 18, Pyruvate; 19, Citrate; 20, Polyunsaturated fatty acid; 21, Asparagine; 22, Lysine; 23, α-Ketoglutarate; 24, Creatine; 25, Creatinine; 26, Choline; 27, Phosphocholine (PC) / Glycerophosphocholine (GPC); 28, Glucose; 29, TMAO (Trimethylamine-N-oxide); 30, Betaine; 31, Glycine; 32, Myo-inositol; 33, Glycerol; 34, Serine; 35, β-glucose; 36, α-glucose; 37, Urea; 38, Tyrosine; 39, Histidine; 40, Phenylalanine; 41, Formate.

**Fig 2:**
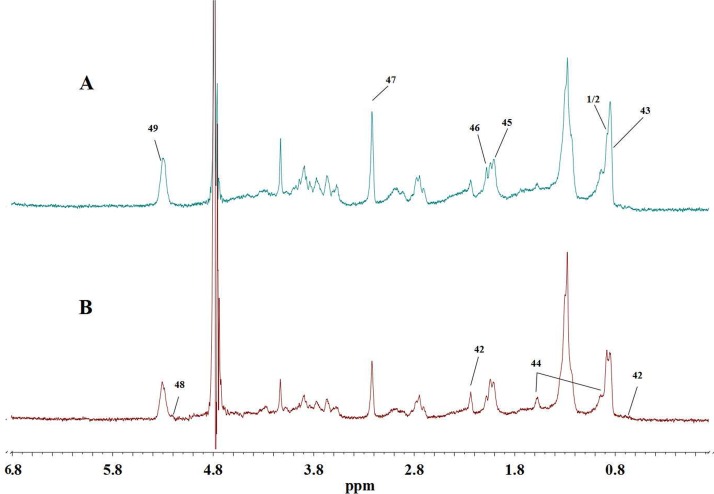
Typical 600 MHz ^1^H BPP-LED spectra of serum samples. (A) controls (B) tumor-bearing mice. Keys for metabolites: 42, Cholesterol; 43, Lipids (mainly HDL); 44, Lipids (triglycerides and fatty acids); 45, O-acetyl glycoproteins; 46, Glycerolipids; 47, Phosphatidylcholine; 48, Triglyceride; 49, Unsaturated lipid.

Serum comprises both low molecular weight metabolites and high molecular weight proteins and lipoproteins. To amplify the low weight metabolites in serum samples, the CPMG plus sequence was employed to acquire the spectra. Typical CPMG spectra of serum samples for controls and tumor-bearing mice are shown in Fig 1. Based on the literature [25–28], the software AMIX (v 3.9.12, Bruker BioSpin) and the Human Metabolome Database [29], major metabolites in serum were identified. The results were further confirmed with two-dimensional NMR data. The spectra of BPP-LED plus sequence is shown in Fig 2, presenting only broad peaks from the lipids and glycoproteins. ^1^H MNR data and assignments for the metabolites in serum are shown in supplementary (S1 Table).

### Multivariate analysis and cross validation

**Fig 3:**
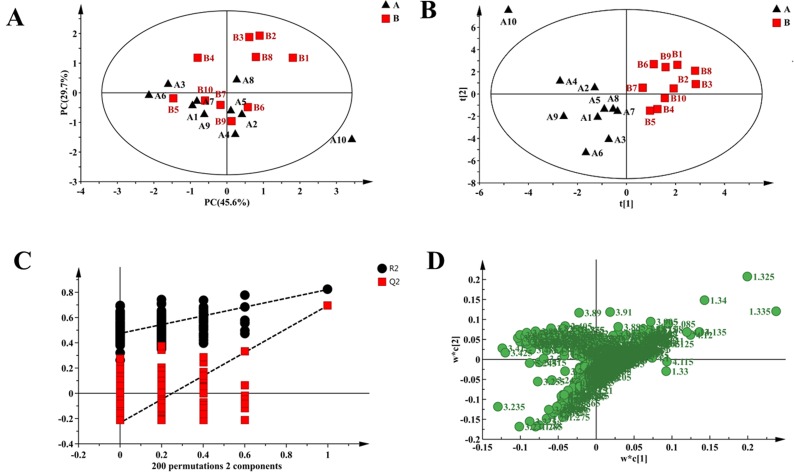
Multivariate analysis of CPMG spectra of serum samples of control and tumor—bearing mice. (A) The score scatter plot of PCA for controls (black triangle) and tumor-bearing mice (red box). (B) PLS-DA showed a clear separation between controls (black triangle) and tumor-bearing mice (red box) in the score scatter plot. (C) Permutation test results for PLS-DA models (R2 = (0.0, 0.482), Q2 = (0.0, -0.214)). (D) Loading plot corresponding to PLS-DA score scatter plot.

Multivariate analysis of ^1^H NMR spectra was used to screen the different metabolites between the controls and tumor-bearing mice. At first, unsupervised PCA was used to analyze the ^1^H NMR CPMG spectra. The score scatter plot of PCA followed by examination of the first two principal components failed to reveal any clear separation between controls and tumor-bearing mice (Fig 3A). The score scatter plot of PLS-DA followed by examination of the first two principal components showed clear separation between controls and tumor-bearing mice along with R2X (cum) = 53.9%, R2Y (cum) = 82.2% and Q2 (cum) = 69.6% (Fig 3B). The results from CV-ANOVA (p = 0.0027) and permutation tests (Fig 3C) showed high quality for the PLS-DA model of the CPMG spectra. The VIP > 1 plots of the PLS—DA were considered a greater contribution to the clustering of groups, including statistically significant difference (Mann-Whitney test, p < 0.05) as the main research objective. The loading scatter plot corresponding to PLS-DA score plot (Fig 3D) showed higher levels of isoleucine, leucine, glutamate, citrate, lysine, α-ketoglutarate, glycerol, betaine, glycine, creatine, serine, choline, lactate, tyrosine, phenylalanine, histidine and formate and lower levels of VLDL, unsaturated lipids, glucose, pyruvate and phosphocholine (PC) / glycerophosphocholine (GPC) in the serum samples of tumor-bearing mice than in controls (Table 1).

**Table 1:** Metabolites responsible for the differences between tumor-bearing mice and controls.

Metabolites	δ1H (ppm)	Multiplicity	p-value	Changes in tumor-bearing mice compared to controls
Isoleucine	0.94	t	0.0089	↑
Leucine	0.95	d	0.0355	↑
VLDL	1.26	m	0.0433	↓
Glutamine	2.08	m	<0.0001	↑
Pyruvate	2.41	s	0.0355	↓
Citrate	2.54	d	0.0355	↑
Lysine	3.01	m	0.0433	↑
α-Ketoglutarate	3.02	m	0.0288	↑
Glucose	3.46, 3.47, 3.48	m	0.0147	↓
Glycerol	3.87	m	0.0001	↑
Phosphocholine (PC)/Glycerophosphocholine (GPC)	3.23	s	0.0115	↓
Betaine	3.28	s	0.0232	↑
Glycine	3.56	s	0.0068	↑
Creatine	3.93	s	0.0288	↑
Serine	3.945, 3.95, 3.97	m	0.0147	↑
Choline	3.66	m	0.0007	↑
Lactate	4.12	q	0.0232	↑
α-Glucose	5.24	d	0.0039	↓
Tyrosine	6.9	d	0.0089	↑
Phenylalanine	7.33	m	0.0147	↑
Histidine	7.75	t	0.0029	↑
Formate	8.46	s	0.0147	↑
Unsaturated lipids	5.29	m	0.0115	↓

**Fig 4:**
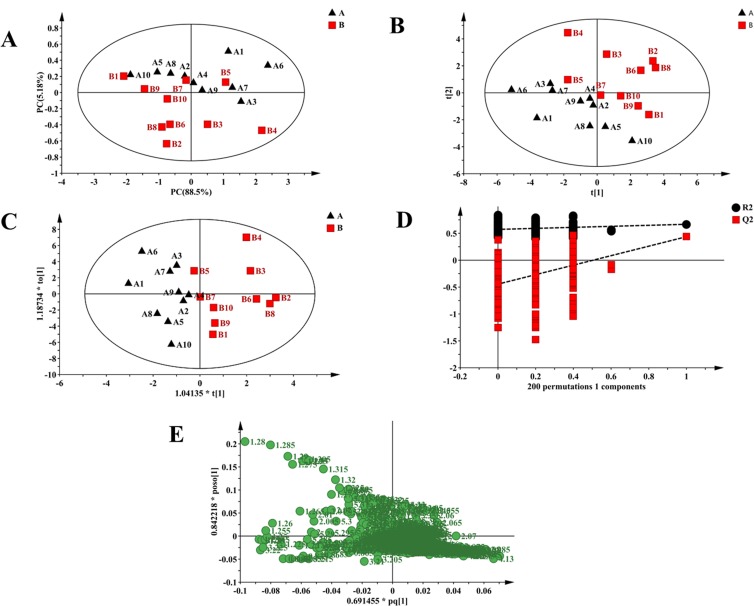
Multivariate analysis of BPP-LED spectra of serum samples in control and tumor-bearing mice. (A) The score scatter plot of PCA for controls (black triangle) and tumor-bearing mice (red box). (B) The score scatter plot of PLS-DA for controls (black triangle) and tumor-bearing mice (red box). (C) OPLS-DA showed clear separation between controls (black triangle) and tumor-bearing mice (red box) in the score scatter plot. (D) Permutation test results for PLS-DA models (R2 = (0.0, 0.56), Q2 = (0.0, -0.404)). (E) Loading plot corresponding to PLS-DA score scatter plot.

The models of PCA and PLS-DA for BPP-LED spectra of serum could not commendably separate controls and tumor-bearing mice (Fig 4A and 4B). Therefore, OPLS-DA was used to analyze the ^1^H NMR BPP-LED spectra. The score scatter plot of OPLS-DA showed clear separation between controls and tumor-bearing mice along with R2X (cum) = 67.5%, R2Y (cum) = 66.7% and Q2 (cum) = 43.0% (Fig 4C). The results from CV-ANOVA (p = 0.05760) and permutation tests (Fig 4D) showed high quality for the OPLS-DA model of the BPP-LED spectra. The VIP > 1 plots of the OPLS-DA were considered a greater contribution to the clustering of groups, including statistically significant difference (Mann-Whitney test, p < 0.05) as the main research objective. The loading scatter plot corresponding to OPLS-DA score plot (Fig 4E) showed a lower level of unsaturated lipids in the serum samples of tumor-bearing mice than in controls (Table 1).

### Hierarchical cluster analysis

**Fig 5:**
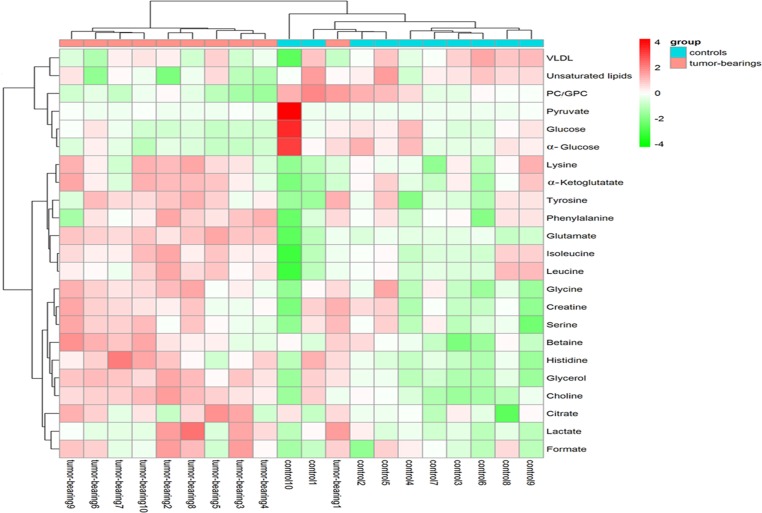
Heat map of the 23 significantly changed serum metabolites in the control and tumor—bearing mice.

To fully and intuitively display the relationships and differences between different samples, HCA (Fig 5) was conducted. The raw data of the heatmap is shown in supplementary (S2 Table). The sample and metabolite differences were simultaneously hierarchically clustered. The horizontal axis of the figure shows a dendrogram of the samples. The samples of the tumor-bearing mice and controls were clustered together. The concentrations of different metabolites varied significantly between tumor-bearing mice and controls. The vertical axis of the figure shows a dendrogram of the metabolite differences. Obvious clustering and relevance of the metabolite differences is shown in the HCA. The metabolites in the same or similar metabolic pathways were clustered together first. For instance, VLDL and unsaturated lipids belonging to the fatty acid metabolism pathway were first clustered together; phenylalanine, tyrosine and glutamate used in anaplerosis in the TCA cycle were also first clustered together. Heatmap which was centered and scaled to the data in the sample direction is shown in supplementary (S1 Fig).

### Investigation of the diagnostic potential of metabolite differences

**Fig 6:**
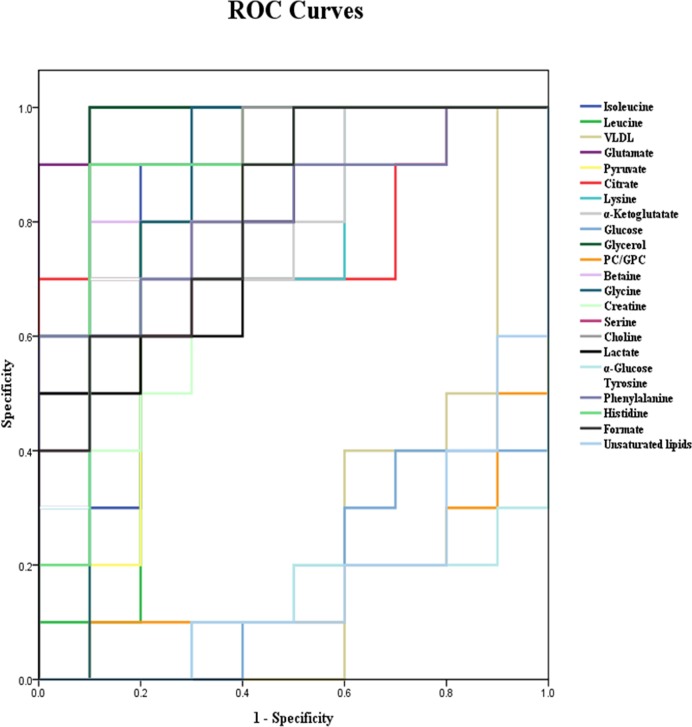
ROC curves for distinguishing controls from tumor-bearing mice according to metabolite differences.

The ROC curves based on the result of area under the curve (AUC) can be conducted to investigate the clinical diagnostic potentials of these significantly different metabolites. The diagnostic accuracy is higher when the value of the AUC is closer to 1. As shown in Fig 6, the AUC of isoleucine (0.84), leucine (0.78), pyruvate (0.78), lysine (0.77), α-ketoglutarate (0.79), betaine (0.87), glycine (0.85), creatine (0.79), serine (0.82), lactate (0.80), tyrosine (0.84), phenylalanine (0.82), histidine (0.88) and formate (0.82) were in the range of 0.7 to 0.9, indicating diagnostic accuracy. Glutamate (0.99), glycerol (0.96) and choline (0.92) had a higher diagnostic accuracy owing to their AUC greater than 0.9. VLDL (0.23), unsaturated lipids (0.17), PC/GPC (0.17), glucose (0.18), and α-glucose (0.13) did not demonstrate diagnostic accuracy because the values of the AUC were less than 0.5.

## Discussion

Serum metabolomics are thought to be a collective "snapshot" of changes throughout the body's metabolism. The alterations of serum metabolomics may be induced by many factors, such as disease, behavior, gender, drug intake, and environmental factors. However, when these factors are similar, the differences of serum metabolomics between the tumor patients and the healthy controls may be derived from the presence of tumor cells. For instance, DA MacIntyre et al. found that the serum pyruvate and glutamate levels may be a good indicator of chronic lymphocytic leukemia (CLL) patients [18]. These authors hypothesized that increased serum levels of pyruvate and glutamate may result from the metabolic changes of the pyruvate kinase type M2 in tumor cells [18]. In a study of hepatocellular carcinoma (HCC) patients by serum metabolomics, Zeng et al. found that the alteration of cancer cell metabolism may be the source of the differences in serum metabolites between healthy controls and HCC patients [30].

In this study, we analyzed the differences of serum metabolite levels in BL mice and wild-type mice based on NMR-based metabolomics. Although the changes of serum metabolites may be associated with many factors such as behavior, gender and environment, these factors were almost identical in our tumor-bearing mice and control mice. The only difference between the tumor-bearing mice and the controls was the presence of tumor cells. Therefore, we infer that the changes of metabolite levels in serum may be derived from multiple tumor-related metabolic pathways, involving energy metabolism, amino acid metabolism, fatty acid metabolism and choline phospholipid metabolism.

### Energy metabolism

Different levels of serum metabolites between tumor-bearing BL mice and the control wild-type mice can reflect the changes in energy metabolism. Glucose is a starting material of glycolysis and lactate is an end product of glycolysis. In our study, lower serum glucose levels and higher serum lactate levels in tumor-bearing mice suggested that blood glucose may be rapidly consumed by glycolysis for tumor cell proliferation and growth. This phenomenon is consistent with the “Warburg effect” [31] that tumor cells rely preferentially on glycolysis, which increases glucose consumption and lactate production. This phenomenon has been reported in many tumors, such as human breast tumors [32] and hepatic carcinoma [33]. Thus, we believe that increased lactate and decreased glucose may be explained by the aerobic glycolysis of tumor cells in the blood.

Creatine is a nitrogen-containing organic acid, which can provide substrates for energy and protein synthesis to meet the requirements for cancer cell proliferation [34]. The creatine level was elevated in the serum of tumor-bearing mice in our study. This is presumably because BL requires substantial substrate to support its rapid growth rate, and is consistent with observations in other types of tumor, such as oral squamous cell carcinoma [35] and head and neck squamous cell carcinoma [36].

### Amino acid metabolism

Amino acid metabolism is the basis of life activities. The amino acids in serum can be used to not only feed tricarboxylic acid cycle (TCA cycle) but also provide amino acids for tumor proliferation and growth [37]. The host's amino acid metabolism is disordered because the dynamics of tumor cells are changed. Studies have shown that the amino acid metabolism is specific to tumor cells [38].

In our study, pyruvate was lower in the serum of tumor-bearing mice. Pyruvate is not only an important product of glycolysis but also a starting material of the TCA cycle. Citrate and α-ketoglutarate are intermediate products of the TCA cycle. In our research, lower pyruvate and higher citrate and α-ketoglutarate levels in BL tumor-bearing mice than in controls suggested that the TCA cycle may be altered to cause the accumulation of intermediate products of the TCA cycle.

We observed increased levels of glutamine in our study. Glutamine is a nitrogen carrier and a major fuel substrate for the proliferation of tumor cells. It can enter the TCA cycle after catabolism as the preferred amino acid to provide ATP for tumor cells and it can also be used in the biosynthesis of nucleotides for the tumor cell proliferation [39]. Oxidative damage is the main cause for cells apoptosis, in response to which tumor cells may increase the concentration of the antioxidant metabolites. Glutamate, a metabolic product of glutamine, is the precursor of glutathione (GSH), a critical antioxidant and free radical scavenger for cell survival in tumors [40]. The increased serum levels of glutamine can provide sufficient raw material for the synthesis of glutathione to protect tumor cells from oxidative damage and apoptosis. Moreover, tumor cells can take advantage of glutaminolysis, which provides sufficient anaplerotic flux and produces glutamate and α-ketoglutarate to support TCA cycle. The finding of increased glutamine in serum of tumor-bearing mice is agreement with reports in hepatocellular carcinoma and renal cell carcinoma [12,41]. Similarly, glycine, a precursor of GSH, is also increased, which can increase the synthesis of GSH and provide adequate carbon source for the biosynthesis of purine and pyrimidine in tumor cells. Glycine and methyl-THF, which are important intermediates of the purine and pyrimidine nucleotides for the proliferation of tumor cells, can be obtained through the conversion of serine, which is synthesized from glycolytic intermediate 3-phosphoglycerate [42,43]. Elevated serum serine ensures the supply of glycine and methyl–THF, as first demonstrated in human colorectal cancer [44].

The serum levels for isoleucine, leucine, phenylalanine, tyrosine and histidine, which are essential amino acids, were higher in tumor-bearing BL mice than in the control wild-type mice. Among these amino acids, isoleucine and leucine belong to the branched chain amino acids, which are increased because of the interaction between different amino acid pools in the tumor host [45]. The branched chain amino acids are used for anaplerosis in the TCA cycle in tumor proliferation and growth. Similarly, the conversion of phenylalanine, tyrosine and histidine into fumarate is also used for anaplerosis. Essential amino acids that can only be obtained from outside sources are necessary for the tumor cell growth and metabolism. If the levels of essential amino acids are insufficient, the rapid proliferation of tumor cells will be hampered.

### Lipid metabolism

The levels of VLDL and unsaturated lipids are lower in the serum of tumor-bearing mice. According to this result, the fatty acid metabolism is also increased in tumor cells and the oxidation of fatty acids can deliver bioenergy for tumor cell proliferation and tumor growth [46]. Elevated membrane biosynthesis of tumor cells is likely to utilize a large amount of lipids, resulting in a decrease in serum lipids. The increased triglyceride metabolism results in the elevated levels of glycerol and fatty acids, which can be converted to acetyl-CoA feeding TCA cycle by β-oxidation. Similar observations have been made in renal cell carcinoma [41] and lymphoblastic leukemia [47].

### Choline phospholipid metabolism

Choline plays important roles in choline-mediated one-carbon metabolism and signaling functions of cell membranes [48]. Choline and its derivatives are key metabolites for choline phospholipid metabolism, and abnormal choline phospholipid metabolism in tumors had been reported [49]. In this study, higher levels of choline and lower levels of PC/GPC were observed in the serum of tumor-bearing mice. It is probably because the rapid proliferation of the tumor cells accelerates the demand for choline, and the host may gain more free choline through a variety of sources to meet this demand. The levels of choline and its derivatives are increased in tumor cells and solid tumors [49–51]. Although this conclusion is different from our result, it is not surprising because different tumor cells have different metabolic behavior. Higher GPC and lower PC were reported in breast and ovarian cancers, and the lower GPC and high PC can occur with malignant transformation [52,53]. Choline dehydrogenase catalyzes the oxidation of choline to betaine, which is a key step in choline-mediated one-carbon metabolism [54]. Elevated levels of betaine in the serum of tumor-bearing mice are likely to be caused by changes in choline metabolism.

### Diagnostic potential of metabolite differences

We further tested the diagnostic potential of metabolite differences for diagnosing BL using ROC curves (Fig 6). The result indicated that glutamate, glycerol and choline with AUC values of the ROC curve greater than 0.90 can effectively distinguish tumor-bearing mice from controls. Isoleucine, leucine, pyruvate, lysine, α-ketoglutarate, betaine, glycine, creatine, serine, lactate, tyrosine, phenylalanine, histidine and formate with AUC value of the ROC curve in the range of 0.7 to 0.9 offer diagnostic accuracy in distinguishing tumor-bearing mice from controls. We believe that all of the metabolite differences between the tumor-bearing mice and controls can potentially be used as noninvasive diagnostic biomarkers for BL in the future.

## Conclusions

In summary, the results of this study offer evidence for changes in serum metabolite profiles in a BL mouse model utilizing NMR-based serum metabolomics. Energy metabolism, amino acid metabolism, fatty acid metabolism and choline phospholipid metabolism are altered in BL mice. The diagnostic potential of the metabolite differences was investigated using ROC curves. The results show that glutamate, glycerol and choline had the highest diagnostic accuracy and isoleucine, leucine, pyruvate, lysine, α-ketoglutarate, betaine, glycine, creatine, serine, lactate, tyrosine, phenylalanine, histidine and formate also offered diagnostic accuracy in distinguishing BL mice from wild-type mice. Abnormal metabolism and relevant metabolite differences provide useful clues for developing novel noninvasive methods for the diagnosis and prognosis of BL based on these potential biomarkers.

## Supporting Information

S1 Fig Heatmap of the 23 significantly changed serum metabolites in the control and tumor-bearing mice (centered and scaled the data in sample direction). (TIF)

S1 Table ^1^H MNR Data and Assignments for the Metabolites in Serum. (DOCX)

S2 Table The raw data of the heatmap (C1~C10 as controls; T1~T10 as tumor-bearing mice). (DOCX)
