# Supplementary figures and images for: Serum Metabolomics of Burkitt Lymphoma Mouse Models
Source: PLoS One. 2017 Jan 27;12(1):e0170896. doi: 10.1371/journal.pone.0170896 (PMC5271368; doi:10.1371/journal.pone.0170896)

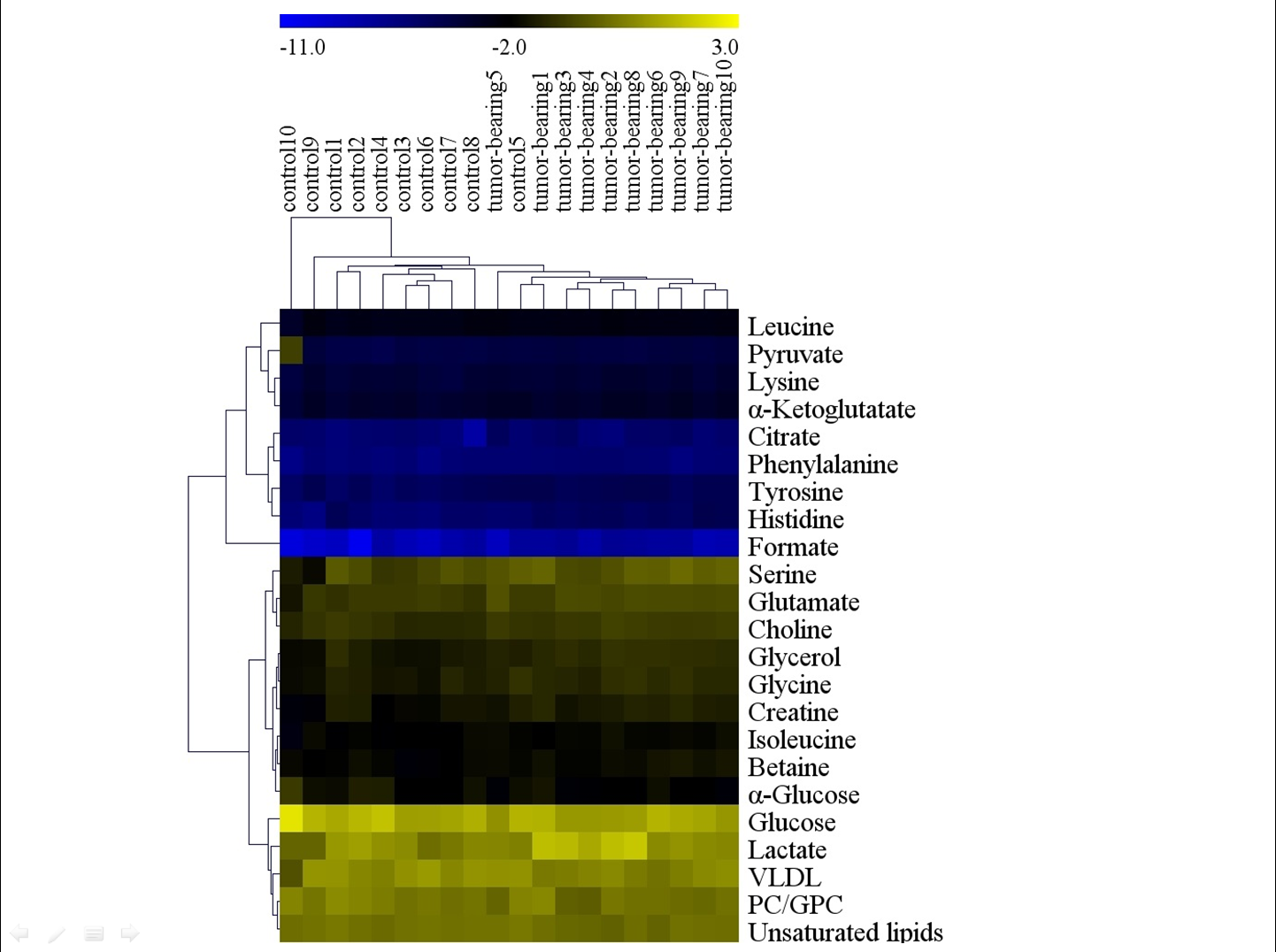

Supplement: S1 Fig — (TIF) [file pone.0170896.s001.tif]
