# Supplementary material for: Serum Metabolomics of Burkitt Lymphoma Mouse Models
Source: PLoS One. 2017 Jan 27;12(1):e0170896. doi: 10.1371/journal.pone.0170896 (PMC5271368; doi:10.1371/journal.pone.0170896)
Supplement: S1 Table — (DOCX) [file pone.0170896.s002.docx]

**S1 Table. ^1^H MNR Data and Assignments for the Metabolites in Serum**

|  | Metabolites | Moieties | Chemical Shifts  (δ^1^H(ppm) and multiplicity) |
| --- | --- | --- | --- |
| 1 | Lipids(mainly LDL) | CH3(CH2)n, (CH2)n | 0.85(t), 1.23(m) |
| 2 | Lipids(mainlyV LDL) | CH3(CH2)n, (CH2)n | 0.88(t), 1.28(m) |
| 3 | Isoleucine | αCH3, βCH3 | 0.94(t), 1.02(d) |
| 4 | Leucine | δCH3, βCH3,βCH2,γCH | 0.92(d), 0.95(d), 1.70(m) |
| 5 | Valine | γ-CH3,αCH, βCH | 0.99(d), 1.05(d), |
| 6 | 3-Hydroxybutyrate | **γ**CH3, **α**CH2 | 1.21(d),2.39(m) |
| 7 | Unknow |  | 1.23(m) |
| 8 | Lactate | βCH3, αCH | 1.33(d), 4.12(q) |
| 9 | Alanine | CH3, αCH | 1.49(d), 3.78(q) |
| 10 | Citrulline | γCH2, βCH2 | 1.58(m) |
| 11 | Arginine | βCH2,γCH2 | 1.89(m),1.73(m) |
| 12 | Acetate | CH3 | 1.92(s) |
| 13 | Proline | γCH2 | 2.00(m) |
| 14 | Glutamate | half-βCH2, γCH2, αCH | 2.08(m),2.12(m),2.34(m),3.75(m) |
| 15 | Glutmaine | βCH2, γCH2, αCH | 2.13(m), 2.45(m), 3.77(m) |
| 16 | Methionine | δCH3, γCH2,βCH2, | 2.14(s),3.78(d,d) |
| 17 | Lipid |  | 2.24(m) |
| 18 | Pyruvate | CH3 | 2.41(s) |
| 19 | Citrate | half-CH2,half-CH2 | 2.54(d) |
| 20 | Polyunsaturated fatty acid | C=C-CH2-C=C | 2.78(m) |
| 21 | Asparagine | half-βCH2, αCH | 2.68(m),2.81(m),3.90(m) |
| 22 | Lysine | αCH2,βCH2,γCH2,δCH2 | 3.76(t),1.89(m),1.72(m),3.01(m) |
| 23 | α-Ketoglutarate | CH2,CH2 | 2.45(t),3.02(m) |
| 24 | Creatine | N-CH3, CH2 | 3.04(s), 3.93(s) |
| 25 | Creatinine | N-CH3, CH2 | 3.04(s),4.05(s) |
| 26 | Choline | N-(CH3)3,NCH2,CH2OH | 3.2(s),3.66(m), 4.30(m) |
| 27 | Phosphocholine (PC) / Glycerophosphocholine (GPC) | N-(CH3)3，OCH2,N-CH2 | 3.23(s)，3.61(t),3.68(t) 4.63（m) |
| 28 | Glucose | H1 | 3.4-4.0 |
| 29 | Trimethylamine-N-oxide | N-(CH3)3 | 3.26(s) |
| 30 | Betaine | N-(CH3)3,N-CH2 | 3.28(s)，3.90(s) |
| 31 | Glycine | CH2 | 3.56(s) |
| 32 | Myo-inositol | 5-CH,4,6-CH,1,3-CH,2-CH | 3.35(m) , 3.63(m), 3.54(dd), 4.07(m) |
| 33 | Glycerol | half-CH2, C2-H | 3.57(d), 3.65(d), 3.87(m) |
| 34 | Serine |  | 3.83(dd), 3.90-4.00(m) |
| 35 | β-glucose | 1-CH | 4.66(d) |
| 36 | α-glucose | 1-CH | 5.24(d) |
| 37 | Urea | NH2+NH2 | 5.78(m) |
| 38 | Tyrosine | CH, CH | 6.90(d),7.19(d) |
| 39 | Histidine | 2-CH,4-CH,CH2 | 7.75(t), 7.08(d),6.05(d) |
| 40 | Phenylalanine | Ring-CH | 7.42(m), 7.33(m), 7.35(m) |
| 41 | Formate | CH | 8.46(s) |
| 42 | Cholesterol | CH3(C18(in HDL),C18(in VIDL),C21),CH2COOR | 0.66(m),0.70(m),0.91(m),2.34(m) |
| 43 | Lipids(mainly HDL) | CH3 | 0.84(t),1.22(m) |
| 44 | Lipids(triglycerides and fatty acids) | CH3(CH2)n,CH2CH2CO, | 1.29(m),1.58(m) |
| 45 | O-acetyl glycoproteins | CH3 | 2.06(s) |
| 46 | Glycerolipids | CH2CO | 2.24(m) |
| 47 | phosphatidylcholine | N-(CH3)3 | 3.22(s) |
| 48 | Triglyceride | CH2OCOR | 5.20(m) |
| 49 | Unsaturated lipid | CH2=CH2 | 5.29(m), 5.31(m) |

s: single; d: doublet; t: triplet; q: quartet; m: multiplet; dd: doublet of doublet
