# Supplementary material for: Serum Metabolomics of Burkitt Lymphoma Mouse Models
Source: PLoS One. 2017 Jan 27;12(1):e0170896. doi: 10.1371/journal.pone.0170896 (PMC5271368; doi:10.1371/journal.pone.0170896)
Supplement: S2 Table — (DOCX) [file pone.0170896.s003.docx]

**S2 Table. The raw data of the heatmap (C1~C10 as controls; T1~T10 as tumor-bearing mice)**

|  | **C1** | **C2** | **C3** | **C4** | **C5** | **C6** | **C7** | **C8** | **C9** | **C10** | **T1** | **T2** | **T3** | **T4** | **T5** | **T6** | **T7** | **T8** | **T9** | **T10** |
| --- | --- | --- | --- | --- | --- | --- | --- | --- | --- | --- | --- | --- | --- | --- | --- | --- | --- | --- | --- | --- |
| **Isoleucine** | **0.2298** | **0.2648** | **0.2485** | **0.2363** | **0.2672** | **0.2420** | **0.2463** | **0.2924** | **0.2939** | **0.1633** | **0.2560** | **0.3165** | **0.2755** | **0.2744** | **0.2966** | **0.2715** | **0.2740** | **0.2759** | **0.2860** | **0.3044** |
| **Leucine** | **0.1290** | **0.1503** | **0.1414** | **0.1372** | **0.1529** | **0.1415** | **0.1431** | **0.1735** | **0.1715** | **0.0932** | **0.1483** | **0.1802** | **0.1545** | **0.1591** | **0.1702** | **0.1541** | **0.1471** | **0.1615** | **0.1557** | **0.1674** |
| **VLDL** | **1.8922** | **1.5718** | **1.8241** | **1.4436** | **1.8676** | **2.0550** | **1.5853** | **1.8769** | **1.9117** | **0.7539** | **1.3209** | **1.6396** | **1.3703** | **1.5149** | **1.8115** | **1.1776** | **1.6404** | **1.3409** | **1.4032** | **1.7272** |
| **Glutamate** | **0.4968** | **0.5522** | **0.5568** | **0.5589** | **0.5759** | **0.5889** | **0.5614** | **0.5056** | **0.5359** | **0.3289** | **0.5786** | **0.6619** | **0.7138** | **0.7113** | **0.7679** | **0.6969** | **0.6805** | **0.7132** | **0.6998** | **0.7018** |
| **Pyruvate** | **0.0447** | **0.0434** | **0.0506** | **0.0386** | **0.0510** | **0.0463** | **0.0478** | **0.0467** | **0.0585** | **0.5996** | **0.0534** | **0.0503** | **0.0600** | **0.0521** | **0.0531** | **0.0572** | **0.0536** | **0.0494** | **0.0584** | **0.0608** |
| **Citrate** | **0.0130** | **0.0162** | **0.0188** | **0.0172** | **0.0160** | **0.0154** | **0.0123** | **0.0046** | **0.0177** | **0.0193** | **0.0204** | **0.0125** | **0.0240** | **0.0139** | **0.0261** | **0.0209** | **0.0149** | **0.0199** | **0.0236** | **0.0194** |
| **Lysine** | **0.0584** | **0.0705** | **0.0736** | **0.0674** | **0.0681** | **0.0589** | **0.0522** | **0.0721** | **0.0844** | **0.0518** | **0.0636** | **0.0818** | **0.0756** | **0.0639** | **0.0770** | **0.0734** | **0.0628** | **0.0852** | **0.0848** | **0.0850** |
| **α-Ketoglutarate** | **0.0702** | **0.0909** | **0.0928** | **0.0830** | **0.1006** | **0.0699** | **0.0774** | **0.0887** | **0.1018** | **0.0618** | **0.0799** | **0.1042** | **0.0938** | **0.0836** | **0.1024** | **0.0934** | **0.0813** | **0.1056** | **0.1096** | **0.1070** |
| **Glucose** | **2.4921** | **3.0681** | **2.1882** | **3.5687** | **2.9354** | **2.2102** | **2.3828** | **2.7435** | **2.9817** | **5.6545** | **2.8161** | **2.0664** | **1.9951** | **1.9992** | **1.8150** | **2.9474** | **2.4154** | **2.1636** | **2.5672** | **1.9719** |
| **Glycerol** | **0.4682** | **0.3855** | **0.3085** | **0.3239** | **0.3910** | **0.3109** | **0.3513** | **0.3692** | **0.2934** | **0.2856** | **0.4335** | **0.5091** | **0.4698** | **0.4384** | **0.4123** | **0.4938** | **0.4725** | **0.4871** | **0.4760** | **0.4558** |
| **PC/GPC** | **1.6893** | **1.5411** | **1.1795** | **1.3756** | **1.5207** | **1.2905** | **1.1537** | **1.2210** | **1.2367** | **1.5356** | **1.6282** | **1.2677** | **0.9419** | **0.9060** | **1.0040** | **1.1674** | **1.0459** | **1.1623** | **1.0859** | **1.1909** |
| **Betaine** | **0.2637** | **0.3034** | **0.1998** | **0.2707** | **0.2780** | **0.2197** | **0.2460** | **0.2891** | **0.2445** | **0.2845** | **0.3155** | **0.3003** | **0.2647** | **0.2646** | **0.2924** | **0.3305** | **0.3191** | **0.2939** | **0.3516** | **0.3376** |
| **Glycine** | **0.4128** | **0.3874** | **0.3438** | **0.3311** | **0.5012** | **0.3040** | **0.4205** | **0.3754** | **0.3029** | **0.2896** | **0.4413** | **0.4786** | **0.4193** | **0.3929** | **0.4055** | **0.4524** | **0.4310** | **0.5003** | **0.4919** | **0.4417** |
| **Creatine** | **0.3929** | **0.3806** | **0.2812** | **0.2519** | **0.3955** | **0.2755** | **0.3367** | **0.3338** | **0.2142** | **0.1922** | **0.4326** | **0.3663** | **0.3277** | **0.3530** | **0.3207** | **0.3986** | **0.3900** | **0.4122** | **0.4389** | **0.3727** |
| **Serine** | **0.8592** | **0.7214** | **0.5438** | **0.5270** | **0.9314** | **0.6498** | **0.8020** | **0.7113** | **0.2878** | **0.3688** | **1.0033** | **0.7558** | **0.7009** | **0.6712** | **0.7869** | **0.9156** | **0.9111** | **0.9377** | **1.0732** | **0.9737** |
| **Choline** | **0.5684** | **0.5267** | **0.4263** | **0.4844** | **0.5173** | **0.4369** | **0.4421** | **0.4507** | **0.5084** | **0.4172** | **0.5039** | **0.6287** | **0.5493** | **0.5410** | **0.5666** | **0.5722** | **0.5688** | **0.5955** | **0.5638** | **0.5923** |
| **Lactate** | **1.9889** | **2.2154** | **1.6951** | **1.8063** | **1.4433** | **1.1316** | **1.3040** | **1.5752** | **1.0050** | **1.0014** | **3.3776** | **3.3735** | **3.1596** | **2.4982** | **1.5515** | **1.6746** | **1.6693** | **3.8315** | **1.8726** | **1.5730** |
| **α-Glucose** | **0.2961** | **0.3909** | **0.2475** | **0.3775** | **0.3103** | **0.2537** | **0.2488** | **0.3211** | **0.3036** | **0.5528** | **0.3348** | **0.2466** | **0.2098** | **0.2225** | **0.2065** | **0.3051** | **0.2484** | **0.2421** | **0.2461** | **0.2002** |
| **Tyrosine** | **0.0211** | **0.0297** | **0.0268** | **0.0189** | **0.0371** | **0.0228** | **0.0281** | **0.0335** | **0.0343** | **0.0211** | **0.0388** | **0.0368** | **0.0293** | **0.0327** | **0.0355** | **0.0381** | **0.0347** | **0.0396** | **0.0276** | **0.0353** |
| **Phenylalanine** | **0.0134** | **0.0148** | **0.0149** | **0.0130** | **0.0159** | **0.0101** | **0.0145** | **0.0157** | **0.0158** | **0.0091** | **0.0164** | **0.0182** | **0.0172** | **0.0179** | **0.0159** | **0.0158** | **0.0148** | **0.0169** | **0.0114** | **0.0156** |
| **Histidine** | **0.0315** | **0.0212** | **0.0155** | **0.0153** | **0.0180** | **0.0137** | **0.0199** | **0.0206** | **0.0102** | **0.0143** | **0.0267** | **0.0292** | **0.0230** | **0.0279** | **0.0174** | **0.0285** | **0.0377** | **0.0236** | **0.0242** | **0.0338** |
| **Formate** | **0.0022** | **0.0007** | **0.0029** | **0.0046** | **0.0056** | **0.0020** | **0.0038** | **0.0051** | **0.0018** | **0.0011** | **0.0055** | **0.0070** | **0.0070** | **0.0041** | **0.0024** | **0.0055** | **0.0030** | **0.0062** | **0.0057** | **0.0034** |
| **Unsaturated lipids** | **1.2994** | **1.1738** | **1.1890** | **1.0748** | **1.2967** | **1.2257** | **1.1763** | **1.1988** | **1.2026** | **1.1436** | **1.1573** | **0.9483** | **1.0419** | **1.0272** | **1.2083** | **0.9836** | **1.1581** | **1.1261** | **1.1817** | **1.1232** |
